# Benefits of home-based multidisciplinary exercise and supportive care in inoperable non-small cell lung cancer – protocol for a phase II randomised controlled trial

**DOI:** 10.1186/s12885-017-3651-4

**Published:** 2017-09-29

**Authors:** Lara Edbrooke, Sanchia Aranda, Catherine L. Granger, Christine F. McDonald, Mei Krishnasamy, Linda Mileshkin, Louis Irving, Sabine Braat, Ross A. Clark, Ian Gordon, Linda Denehy

**Affiliations:** 1 Department of Physiotherapy, The University of Melbourne, Level 7, 161 Barry St, Parkville, VIC 3010 Australia; 2 Cancer Council Australia, Sydney, NSW Australia; 3 Department of Nursing, The University of Melbourne, Parkville, VIC Australia; 4 Institute for Breathing and Sleep, Heidelberg, VIC Australia; 5 Department of Physiotherapy, Melbourne Health, Parkville, VIC Australia; 6 Department of Respiratory and Sleep Medicine, Austin Health, Heidelberg, VIC Australia; 7 The University of Melbourne Centre for Cancer Research, Parkville, VIC Australia; 8 The Peter MacCallum Cancer Centre, Melbourne, VIC Australia; 9 Department of Respiratory and Sleep Medicine, Melbourne Health, Parkville, VIC Australia; 10 Melbourne School of Population and Global Health, The University of Melbourne, Parkville, VIC Australia; 11 University of the Sunshine Coast, Sunshine Coast, QLD Australia; 12 Statistical Consulting Centre, The University of Melbourne, Parkville, VIC Australia

**Keywords:** Non-small cell lung cancer, Home-based exercise, Symptom control, Supportive care, Physical function

## Abstract

**Background:**

Lung cancer is one of the most commonly diagnosed cancers, and is a leading cause of cancer mortality world-wide. Due to lack of early specific symptoms, the majority of patients present with advanced, inoperable disease and five-year relative survival across all stages of non-small cell lung cancer (NSCLC) is 14%. People with lung cancer also report higher levels of symptom distress than those with other forms of cancer. Several benefits for survival and patient reported outcomes are reported from physical activity and exercise in other tumour groups. We report the protocol for a study investigating the benefits of exercise, behaviour change and symptom self-management for patients with recently diagnosed, inoperable, NSCLC.

**Methods:**

This multi-site, parallel-group, assessor-blinded randomised controlled trial, powered for superiority, aims to assess functional and patient-reported outcomes of a multi-disciplinary, home-based exercise and supportive care program for people commencing treatment. Ninety-two participants are being recruited from three tertiary-care hospitals in Melbourne, Australia. Following baseline testing, participants are randomised using concealed allocation, to receive either: a) 8 weeks of home-based exercise (comprising an individualised endurance and resistance exercise program and behaviour change coaching) and nurse-delivered symptom self-management intervention or b) usual care. The primary outcome is the between-group difference in the change in functional exercise capacity (six-minute walk distance) from baseline to post-program assessment. Secondary outcomes include: objective and self-reported physical activity levels, physical activity self-efficacy, behavioural regulation of motivation to exercise and resilience, muscle strength (quadriceps and grip), health-related quality of life, anxiety and depression and symptom interference.

**Discussion:**

There is a lack of evidence regarding the benefit of exercise intervention for people with NSCLC, particularly in those with inoperable disease receiving treatment. This trial will contribute to evidence currently being generated in national and international trials by implementing and evaluating a home-based program including three components not yet combined in previous research, for people with inoperable NSCLC receiving active treatment and involving longer-term follow-up of outcomes. This trial is ongoing and currently recruiting.

**Trial registration:**

This trial was prospectively registered on the Australian New Zealand Clinical Trials Registry (ACTRN12614001268639: (4/12/14).

**Electronic supplementary material:**

The online version of this article (10.1186/s12885-017-3651-4) contains supplementary material, which is available to authorized users.

## Background

Approximately 1.8 million new cases of lung cancer were diagnosed globally in 2012, making it the most frequently diagnosed cancer in males and third most frequently diagnosed in females, after breast and colorectal cancer. In Australia lung cancer is the fifth most commonly diagnosed cancer but the number one cause of cancer mortality [1]. Eighty-five percent of all new diagnoses are non-small cell lung cancers (NSCLC) [2]. Due to a lack of early specific symptoms, people with NSCLC often present when their disease is inoperable, with 52% having metastatic disease at diagnosis [2].

Physical activity (PA) is defined as ‘any bodily movement produced by skeletal muscle that requires energy expenditure’ [3]. Several health benefits of increased PA are reported for cancer populations [4–7]. The American College of Sports Medicine (ACSM) recommendations for PA in cancer survivors are in line with current recommendations for the healthy population; avoid sedentary time, and perform at least 150 min of moderate-intensity (or 75 min vigorous-intensity) aerobic exercise and two-to-three resistance-training sessions per week [8]. Increased PA is shown to be associated with improved survival, predominantly in breast (13 studies reported a decrease in breast cancer mortality of between 13 and 51% when comparing those in the highest versus lowest PA categories) and colorectal cancers (three studies reported reductions in risk of death from colorectal cancer of between 45 and 61% when comparing those in the highest versus lowest PA categories) [4]. The PA dosage required to attain health benefits across these cancer groups is not uniform, nor is it clear whether results demonstrated can be applied to other cancer populations [4, 5, 9]. In a study of healthy older adults, even low doses of moderate-intensity PA were associated with a 22% (risk ratio 0.78, 95% CI 0.71–0.88, *p* < 0.0001) reduction in mortality risk, compared to those who were inactive [10]. However 82% of UK cancer survivors [11] and 60% of patients with NSCLC do not meet current PA guidelines at diagnosis [12].

Exercise is a subcategory of PA defined as PA that is ‘planned, structured, repetitive, and purposive in the sense that improvement or maintenance of one or more components of physical fitness is an objective’ [3]. A Cochrane systematic review of randomised and quasi-randomised controlled trials concluded that exercise interventions for people with cancer may lead to improved overall health-related quality of life (HRQoL), fatigue and physical and social functioning. Benefits were greatest for moderate-vigorous exercise compared to mild exercise programs [13]. Moderate-intensity exercise (70% intensity) is reported to be most effective in improving walking endurance in mixed tumour groups [14]. In those with advanced NSCLC, preliminary evidence from prospective cohort studies demonstrates that higher functional exercise capacity, as measured by six-minute walk distance (6MWD), predicts improved survival and slower disease progression [15, 16].

In those with advanced NSCLC, a systematic review of exercise interventions from two studies reported improvements in well-being and symptoms in participants who were adherent to moderate-intensity exercise programs [17]. Exercise interventions in people predominantly receiving curative surgical intervention for NSCLC, report these programs to be safe, and preliminary data demonstrate improvements in exercise capacity (peak oxygen uptake (VO_2_ peak) and 6MWD), muscle strength and cancer-related fatigue immediately post-program with few adverse events [18]. The sole study identified in the systematic review involving people with advanced NSCLC [19], demonstrated a significant reduction in symptoms, maintenance of exercise capacity and muscle strength on completion of an eight-week hospital-based exercise program. Despite this growing body of evidence for the benefits of exercise, current international guidelines do not include recommendations regarding exercise prescription in the care of people with advanced NSCLC [20].

Investigation of the benefits of exercise intervention during treatment for inoperable NSCLC is important as such interventions may result in fewer treatment side-effects and better treatment tolerance, physical function and health related quality of life (HRQoL). Improvements in functional exercise capacity and HRQOL are reported following supervised exercise interventions for patients with advanced NSCLC undergoing chemotherapy in both inpatient and outpatient hospital settings [21–24] as well as for those receiving targeted therapy [25]. Of note, the randomised controlled trials (RCTs) performed to date with this group of patients are underpowered [21, 22, 25] and no study has followed patients beyond program completion.

A number of RCTs are currently being conducted involving people with inoperable lung cancer, mostly receiving palliative treatment. These studies are largely hospital-based [26–29], are powered to detect outcomes other than functional exercise capacity [28, 30] and in one study including those receiving curative intent treatment, exercise does not commence until at least 4 weeks *after* radical chemo/radiotherapy completion [30]. Our study will address gaps in the current evidence base for exercise intervention by implementing a multi-disciplinary, solely home-based exercise and supportive care intervention, during active treatment for people with inoperable NSCLC and including six-month follow up of participants.

The primary aim of this study is to assess the efficacy of home-based, multi-disciplinary exercise and supportive care, on change in functional exercise capacity (6MWD) in people with inoperable NSCLC. The intervention comprises a package of care incorporating exercise, symptom management and behavior change techniques. We hypothesise that those receiving the exercise and supportive care program will have a smaller decline in functional exercise capacity from baseline to nine-weeks, compared to participants receiving usual care. Key secondary aims are to assess whether multi-disciplinary, home-based exercise and supportive care, is superior to usual care for patient-reported and performance-based outcomes including physical activity and muscle strength from baseline to nine-weeks. Semi-structured interviews will be undertaken with a subgroup of intervention participants to understand the participant experience of involvement in the program. Exploratory outcomes aim to explore differences in 3-year survival, muscle ultrasound and inflammatory markers between groups in a subset of patients measured.

## Methods/design

### Study design and setting

This two-arm, parallel (1:1), superiority, assessor-blinded, randomised controlled trial is being conducted at three hospitals within Melbourne, Australia that form part of the Victorian Comprehensive Cancer Centre Alliance (The Peter MacCallum Cancer Centre (MK site principal investigator), the Royal Melbourne Hospital (LI site principal investigator) and The Austin Hospital (CM site principal investigator)). Study chief investigators (LD, SA, CM, MK, LI, LM, RC) meet biannually with the study co-ordinator (LE) to review study procedures and progress. The reporting of this randomised controlled trial protocol follows Consolidated Standards of Reporting Trials (CONSORT) [31], Standard Protocol Items: Recommendations for Interventional Trials (SPIRIT) [32] and Template for Intervention Description and Replication (TIDier) [33] guidelines. This trial has been prospectively registered on the Australian New Zealand Clinical Trials Registry (http://www.anzctr.org.au): ACTRN12614001268639. Recruitment commenced in December 2014.

### Participants

**Fig. 1:**
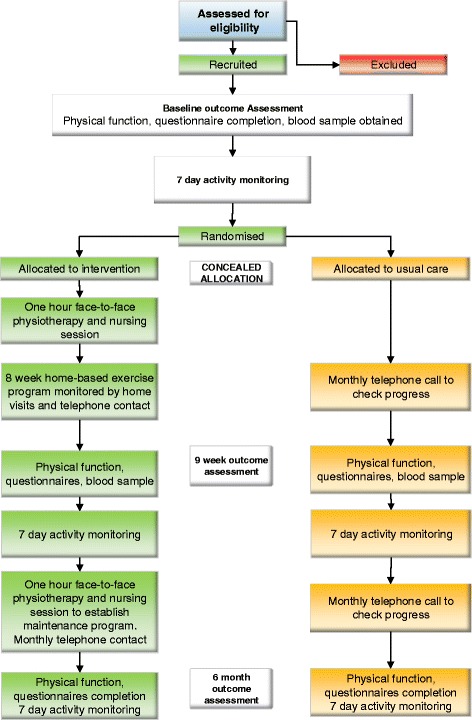
Participant flow through the trial

Figure 1 outlines participant flow through the study. Eligible participants at each of the participating sites are identified through screening outpatient lung oncology clinic lists, discussions at lung oncology multi-disciplinary team meetings and inpatient admissions. To be eligible participants must have a diagnosis of inoperable NSCLC, be scheduled to receive treatment for the primary lung tumour other than surgery (ie: chemotherapy, radiotherapy or targeted therapy), have commenced treatment no more than 4 weeks prior to recruitment, be aged ≥ 18 years, be able to read and write English, have an Eastern Co-operative Oncology Group (ECOG) performance status [34] of ≤ two and a Clinical Frailty Scale (CFS) score [35] of < seven; have a physician rated life expectancy > 6 months and the treating oncologist’s approval for study involvement. Participants are excluded if they have a concurrent, actively treated other malignancy (or one-year history of other malignancy (three-years for breast cancer due to proximity of the radiotherapy treatment field to the lung)) other than non-melanoma skin cancer or in-situ melanoma, any co-morbidities or evidence of pelvic or lower limb bony metastases prohibiting participation in a land-based exercise program, met PA guidelines in the past month based on self-report (150 min or more of moderate intensity PA per week), or have a current unstable psychiatric or cognitive disorder.

Eligible participants are contacted by the trial co-ordinator who explains the study aims and requirements, and those expressing interest in the study are provided with a Patient Information and Consent Form (Additional file 1: Appendix 1). All participants provide written informed consent prior to completing baseline outcome measures. Recruited participants may choose to withdraw from the study at any stage. Data collected prior to the time of study withdrawal will be included in data analyses.

### Randomisation and allocation

Following informed consent and all baseline assessments, participants are randomised 1:1 to either the intervention group (exercise and supportive care) or the control group (usual care). A stratified block permuted randomisation is used with hospital and cancer treatment intent (‘radical’ versus ‘palliative’) as the stratification factors, to ensure balance in treatment assignment within these hospital and cancer treatment intent groups. The randomisation schedule was prepared by a researcher independent of the trial. Consecutively numbered, sealed opaque envelopes are kept in a locked location and distributed by personnel not involved in the trial.

The trial is being conducted in accordance with the Declaration of Helsinki and has undergone multi-site ethics review by the Peter MacCallum Cancer Centre Human Research Ethics Committee and received approval 26/6/2014 (HREC/14/PMCC/27).

### Intervention; phase one (weeks one-eight)

Prior to the first session, the physiotherapist uses Google Maps to assess the terrain surrounding the participant’s house and determine a suitable flat walking track. Scripted sessions incorporate an element of behaviour change, with all physiotherapists delivering the intervention trained in the methods used by Health Change Australia™. This involves a health coaching approach where participants work collaboratively with their physiotherapist to change their exercise behaviours [36]. If participants are receptive, the session commences with education regarding the potential health benefits of exercise for people with cancer. Participants are asked about exercise they have performed previously and during the initial session, the physiotherapist works with the participant to establish an individualised endurance and resistance program to be performed the following week. Potential exercise enablers and barriers are discussed and the physiotherapist works with patients to identify possible strategies to overcome barriers in order that they can complete their exercise prescription. A complete list of behaviour change techniques utilised, as defined by Michie and colleagues’ taxonomy [37], is provided in Table 1. Activity modifiers in this population have previously been identified and include symptoms related to both participant’s disease process and treatment, weather and past activity patterns [38]. Spouse or carer attendance during the first exercise session is encouraged.

**Table 1:** Summary of behavior change techniques

Technique number	Technique
2	Provide information regarding consequences of behaviour to the individual
7	Action planning – detailed planning of what the person will actually do (eg. location, frequency and duration of exercise)
8	Barrier identification/problem solving
16	Self-monitoring of behavioural outcomes – utilising study diary and FitBit
18	Promoting focus on past success
19	Providing feedback on performance
21	Providing instruction on how to perform the behaviour
22	Modelling/demonstrating the behaviour
23	Teach how to use prompts/cues to remind them to perform the behaviour (eg. leaving runners at the front door, SMS exercise reminders)
24	Environmental restructuring
27	Use of follow-up prompts – reduction of contact during the study ‘maintenance’ phase
29	Plan social support/social change
35	Relapse prevention/coping planning – identification of situations where the changed behaviour may not be maintained and planning how to manage these situations
38	Time management – discussing opportunities to exercise, especially during active treatment

### Intervention: exercise

The exercise intervention comprises eight-weeks of weekly individualised endurance and resistance home-based exercises delivered by a physiotherapist. Exercise prescription follows the Frequency, Intensity, Time and Type (F.I.T.T) training principles detailed by Sasso and colleagues [39]. The initial session is conducted face-to-face in the participant’s home with the physiotherapist unless another location is requested by the participant, with weekly follow-up in the form of 10-min telephone sessions. An additional two home-visit exercise sessions may be provided during the eight-week program if deemed necessary by the intervention physiotherapist or at the request of the participant. This may be the case if the participant reports they have been unable to achieve the past week’s exercise goals.

- *Endurance component:* participants are given the option of walking, swimming or cycling. It is anticipated that the majority of participants will choose walking as people with advanced lung cancer have previously expressed a preference for performing walking over other forms of endurance activities [38]. At program commencement, endurance exercises are prescribed for a minimum of 10 min, twice weekly, at a moderate intensity (four to six on the Borg dyspnea scale) [40] depending on initial assessment. If participants are unable to walk for 10 min they are encouraged to walk for shorter durations with increased frequency, aiming to gradually progress duration of endurance exercise to at least 150 min of moderate-intensity exercise per week [8] by completion of the eight-week program.
- *Resistance component:* at the initial session the physiotherapist determines the appropriate resistance required so that the participant can perform only 10 repetitions of each exercise (10 repetition maximum (RM)). Participants are asked to complete 80% of this (eight repetitions), commencing with between one-three sets as per American College of Sports Medicine (ASCM) resistance training guidelines [41]. These exercises are performed at a four to six (somewhat hard) rating of perceived exertion (RPE) [40]. Resistance exercises focus predominantly on functional activities and include: squats, sit-to-stand, heel raises, step-ups, unilateral shoulder elevation, wall press and unilateral shoulder horizontal extension. Participants are provided with hand-weights, based on initial and review assessment findings, and are encouraged to perform resistance exercises every second day.

Each intervention participant receives a DVD demonstrating the resistance exercise program which they are encouraged to review weekly. The DVD also contains a motivational interview with a cancer survivor regarding their experiences of exercising throughout and beyond treatment. Participants are advised not to exercise if they are febrile (above 38.0 C) or have new onset chest pain. Contact numbers for the medical oncology registrar at each hospital site and the study investigators are provided. Participants are also provided with an exercise diary, containing details of their weekly prescribed endurance and resistance exercises and Borg dyspnea and rating of perceived exertion scales. They are asked to record details of endurance and resistance exercises completed each week, along with any issues that need to be raised; diaries are used by the physiotherapist to assess exercise adherence and to progress the exercise program during weekly follow-up telephone sessions. Wherever possible, the participant is encouraged to have their spouse/carer/friend complete the exercise program with them. Exercise programs are progressed when participants have achieved the previous week’s exercise goals and feel they are working below the target RPE. Participant confidence to achieve the progressed exercise program is assessed and only participants who indicate they are confident at a level of seven or above (on a 10-point visual analogue scale (VAS), rated from ‘not confident at all’ to ‘completely confident’) of being able to complete the new program are progressed.

During the program participants are provided with a FitBit Zip™ and encouraged to wear this during waking hours and to record their daily step counts in their exercise diary. Participants also receive daily SMS exercise reminders and are provided with a smartphone if required, to receive these messages.

### Intervention: symptom self-management

Several days following the first exercise session home-visit the trial nurse (an experienced cancer nurse) contacts the patient and provides symptom self-management intervention and advice using the Edmonton Symptom Assessment Scale (ESAS) [42] to standardise each consultation. The ESAS is validated in an oncology population receiving palliative care [43]. Participants are provided with a copy of the ESAS and a symptom self-management education booklet to refer to during nursing consultations. The proactive10-minute nursing consultations continue weekly throughout the phase one eight-week program, with particular emphasis on self-management of symptoms which may be impacting on their ability to exercise. If participants score higher than 4/10, indicating moderate to severe symptom levels [44], for any item of the ESAS the trial co-ordinator is notified and this is followed up with the patient’s medical treating team.

### Intervention adherence

Intervention participants will be defined as adherent to the eight-week program if they complete at least two endurance exercise sessions per week for a minimum of 6 weeks during the eight-week program as has been previously reported in a lung cancer population [23]. A resistance session is defined as complete if a minimum of 50% of prescribed resistance exercises have been performed during that session.

### Maintenance phase (nine weeks – six months)

The maintenance phase commences with a physiotherapy home-visit session to review current exercise programs and work with the participant to progress the program if required (where participants have met exercise goals set in the previous session, are reporting they are now working at less than a ‘somewhat hard’ level and are confident in progressing their exercises). Subsequently, participants are contacted by the physiotherapist by telephone every two to 4 weeks. The frequency of calls is determined by the physiotherapist in consultation with the participant and is based on adherence, motivation and confidence in performing the exercise program during phase one. These scripted sessions employ health coaching techniques to assist participants in setting achievable goals, discuss personalised exercise enablers and barriers, assist in maintaining motivation to continue exercising and improve exercise self-efficacy. To measure adherence to exercise during this phase, participants are asked to record monthly totals for the number of endurance and resistance exercise sessions they have performed. These diaries are returned upon completion of the study.

### Control: usual care

Control group participants receive standard care for people with inoperable NSCLC at each site. Exercise prescription, from either a physiotherapist or exercise physiologist, is currently not standard care in either the inpatient or outpatient settings at any of the study sites. In addition to standard care, to counter the effects of multiple contacts within the intervention group, control group participants receive monthly ‘attention’ phone calls from a study staff member. During these calls, participants are asked about their general well-being. Participants are not given specific advice to increase their PA or exercise levels during these calls. Average number and duration of calls will be reported. Control group participants are offered access to study intervention materials (exercise and symptom self-management booklets, exercise DVD) following completion of their final set of outcome measures.

### Safety and adverse event reporting

A serious adverse event is defined in this study as any event occurring either during or up to 60 min following trial intervention or outcome assessment which is life threatening or results in death, hospitalization or prolongation of existing hospitalization, disability or incapacity [45]. Minor adverse events directly relating to intervention or outcome measure sessions can include: falls not resulting in injury, severe breathlessness, new or progressive pain, neurological deficits, altered mental status, palpitations and progressive fatigue [46]. Following each intervention and outcome measure session trial staff are required to complete data entry forms indicating if a serious or minor adverse event has occurred. In the case of serious adverse events the study chief investigator and trial co-ordinator are notified immediately, participants are managed appropriately and the incident is reported to the relevant hospital ethics committee.

### Outcomes

A summary of study outcomes is provided in Table 2. Outcomes are assessed during a single appointment at baseline, nine-weeks and six-months by research assistants (assessors) blinded to group allocation. At baseline, demographic and clinical details are recorded including age, sex, body mass index (BMI), smoking status, medical history, social history, diagnosis and stage, treatment details and co-morbidities (using the Colinet Comorbidity score) [47] and frailty is assessed at baseline using the Clinical Frailty Scale (CFS) [35]. Survival data are collected until 3-years post study recruitment.

**Table 2:** Summary of outcome measures

	Time point			
Outcomes	Baseline	Post-program (9-weeks)	4-months (telephone)	6-months
Primary outcome				
6MWD	✓	✓		✓
Key secondary outcomes				
Physical Activity				
Accelerometry	✓	✓		✓
IPAQ	✓	✓		✓
Strength				
HHD quadriceps	✓	✓		✓
HGD	✓	✓		✓
Secondary outcomes				
HRQoL				
FACT-L	✓	✓		✓
AQoL	✓	✓		✓
PAAI	✓	✓		✓
BREQ-2	✓	✓		✓
MDASI-LC	✓	✓		✓
HADS	✓	✓		✓
CD-RISC	✓	✓		✓
Health economic questionnaire		✓	✓	✓
Qualitative interviews (subset)		✓		
Exploratory outcomes (subset)				
Venous blood sample	✓	✓		
Quadriceps size and echogenicity	✓	✓		

*6MWD* six minute walk distance, *IPAQ* International Physical Activity Questionnaire, *HHD* hand-held dynamometry, *HGD* handgrip dynamometry, *HRQoL* health-related quality of life, *FACT-L* Functional Assessment of Cancer Therapy-Lung, *AQoL* Assessment of Quality of Life, *PAAI* Physical Activity Assessment Inventory, *BREQ-2* Behavioural Regulation in Exercise Questionnaire Version 2, *MDASI-LC* MD Anderson Symptom Inventory-Lung Cancer, *HADS* Hospital Anxiety and Depression Scale, *CD-RISC* Connor Davidson Resilience Scale. Survival, collected until 3-years, and serious and minor adverse events will be collected until 6-months

Treatment efficacy will be determined by changes in the primary outcome 6MWD from baseline to nine-weeks. This is a commonly used submaximal test of functional exercise capacity [48] that has been found to predict outcomes [49, 50] in patients with lung cancer. The test is being performed according to the American Thoracic Society (ATS) guidelines [50], including duplicate tests to account for the learning effect. Participants are asked to walk for six-minutes on a straight, 30 m track, covering as much distance as possible during this time. Peripheral oxygen saturation (SpO2) is measured continuously throughout the test. Participants are asked to stop walking if SpO_2_ falls below 85% and the test is ceased if SpO_2_ is persistently below this level. The minimal important difference (MID) for decline in 6MWD in lung cancer has been reported to be between 22 and 42 m [51].

Key secondary outcomes relate to PA levels and peripheral muscle strength.

- PA: both objective (motion sensors - Sensewear™ armbands) and patient reported (International Physical Activity Questionnaire – Short Form [IPAQ]) methods are measured. Sensewear™ armbands have been used previously in chronic disease populations [52, 53] and are lightweight, easy to apply and worn on the posterior aspect of the participant’s upper arm. The daily wear time, steps, energy expenditure, metabolic equivalents (METs), sedentary time and time spent in light, moderate and vigorous PA will be reported. The minimum data requirement is 4 days of 8 hours monitoring [54]. The IPAQ asks participants to report on frequency and duration of walking, moderate and vigorous-intensity activities over a seven-day period. PA levels are reported as energy expenditure per week ((METs) minutes/week) for each PA intensity level. The IPAQ has been previously validated [55] in an elderly population and used in cancer populations.
- Muscle strength: is measured using hand-held dynamometry for quadriceps strength (Commander Powertrack II™) and handgrip dynamometry (Jamar™) tested bilaterally, three measures on each side following a practice trial. Hand-held and handgrip dynamometry have been used previously to test quadriceps [56] and handgrip [57] strength in cancer.

#### Secondary outcomes

- Health-related quality of life: measured using the Functional Assessment of Cancer Therapy-Lung (FACT-L) and the Assessment of Quality of Life (AQoL). The FACT-L is a commonly applied 36-item questionnaire containing nine lung cancer specific questions. The FACT-L has demonstrated validity and reliability [58]. The AQoL is a 15-item tool consisting of five domains and provides a utility score used for cost-utility analyses. It is valid and reliable in lung cancer [59].

Physical activity self-efficacy is measured by the Physical Activity Assessment Inventory (PAAI) [60]; a 13-item tool developed to measure self-efficacy for exercise under different conditions summarised into one mean score. The Behavioural Regulation in Exercise Questionnaire (BREQ)-2 is used to assess behavioural regulators of motivation to exercise [61]. It is a 19-item tool consisting of five subscales. An overall score of participant self-determination is derived from the subscales.

Symptom interference is measured using the MD Anderson Symptom Inventory (MDASI-LC) [62] consisting of 16 items on the severity of cancer-related symptoms and six items on the interference with activity, work, walking, mood, relations with others, and enjoyment of life.

The 14-item Hospital Anxiety and Depression Scale (HADS) [63] is used to screen for anxiety and depression symptoms.

Resilience is measured using the 10-item Connor Davidson Resilience Scale (CD-RISC) [64], which has been previously used in a population with breast cancer [65].

Participant experience: qualitative semi-structured interviews are being conducted with a subset of intervention participants following the eight-week program.

Feasibility of delivering the intervention will be measured by recruitment, attrition, and adherence to the program. Additionally, semi-structured interviews will be conducted with the intervention physiotherapists and nurses to gather information regarding feasibility of the intervention.

We are collecting health economic information at nine-weeks and four and six-month time points. This information will be utilized in a health economic analysis run alongside the clinical trial.

#### Exploratory analyses

To answer questions relating to mechanisms underlying our findings, additional measures are being performed in a subset of participants at baseline and nine-weeks only. These include: venous blood samples to assess circulating levels of inflammatory markers in serum and plasma and quadriceps muscle size and echogenicity bilaterally using diagnostic ultrasonography. The samples obtained will be stored at the Department of Respiratory Medicine at the Royal Melbourne hospital until analysis (site prinicipal investigator LI). All samples will be stored securely and confidentially in a laboratory freezer and destroyed at the end of the project according to hospital protocols. Only members of the research team will have access to the blood samples.

### Training and quality

Procedures to ensure data quality and protocol standardisation are in place to minimise bias. These include 1) a detailed intervention and outcome assessment operations manual and 2) face to face training sessions for therapists providing the exercises and assessors at each site with ongoing support from study investigators. To ensure fidelity of trial procedures, therapists at each site meet with investigators experienced in exercise oncology (LD and CG) to review trial process indicators every 3 months. Outcome assessors report episodes of unblinding to the trial co-ordinator and a different, blinded assessor undertakes measures at subsequent time points.

### Sample size calculation

This study is powered to detect a clinically meaningful difference in change in functional exercise capacity (6MWD). Using findings from our pilot sample [66], 32 participants per arm will need to be recruited to detect a between-group clinically relevant mean difference in the 6MWD of 48 m in the change from baseline to nine-weeks with 80% power at a two-tailed 5% level of significance. This assumes an equal standard deviation of 68 m for both groups. After taking into account 30% attrition [66], a total sample size of 92 is required. A sample size >20 is sufficient to determine certainty in qualitative analysis within mixed-methods designs [67].

### Data management and statistical analyses

Study data are being collected and managed using REDCap® electronic data capture tools hosted at the University of Melbourne [68]. REDCap® (Research Electronic Data Capture) is a secure, web-based application designed to support data capture for research studies, providing 1) an intuitive interface for validated data entry; 2) audit trails for tracking data manipulation and export procedures; 3) automated export procedures for seamless data downloads to common statistical packages; and 4) procedures for importing data from external sources. Training of those who collect, check and enter study data will facilitate high quality data, including regular data checks for inconsistency and missing data between and within measurements. Before the start of the statistical analysis, a check will be performed to evaluate the correctness of the randomisation.

The study statisticians were involved in RCT planning and design, will devise a formal detailed statistical analysis plan (including secondary and exploratory analysis) for the study prior to unlocking the data base and will contribute to reporting of results. Baseline characteristics will be summarized by treatment group and imbalances will be investigated. The intention-to-treat principle will apply in all analyses. The primary outcome, the change from baseline to nine-weeks in 6MWD, will be analysed using a mixed-model repeated measures analysis including in the model: baseline, time point, hospital, treatment intent, treatment by time point interaction, and baseline by time point interaction. The primary hypothesis will be examined by a contrast evaluating change from baseline to the nine-week time point in the intervention arm compared to the usual care arm.

Key secondary outcome data (including, motion sensor: steps per day, average energy expenditure, sedentary time; IPAQ: average MET-minutes/week; average quadriceps and grip strength) will be summarized and analysed similarly to the primary outcome. Additional secondary outcome questionnaire data (FACT-L, AQoL, PAAI, BREQ-2, MDASI-LC, HADS and CD-RSC) will be summarized, and will be analysed using either parametric or non-parametric methods depending on the assumptions of the data. A cost- effectiveness analysis will be run alongside the clinical trial and reported separately to the main paper.

Exploratory analyses of 3-year survival will include descriptive Kaplan-Meier survival curves and cox regression with treatment intent, hospital, and cancer treatment type in the model. Subset analyses: circulating levels of inflammatory markers in serum and plasma, quadriceps size and echogenicity bilaterally will be summarized by treatment group and between-group comparisons will be reported.

A per-protocol analysis, for adherent participants as previously defined, will be performed for the primary and key secondary outcomes and the exploratory outcome of survival.

- A priori subgroup analyses: The following subgroups defined using data collected at baseline will be investigated using interaction tests between treatment group and the subgroup variable: a) cancer treatment intent (‘radical’ versus ‘palliative’); b) performance status (ECOG ‘0/1’ versus ‘2’); c) levels of 6MWD based on tertiles of the sample distribution and d) PA based on tertiles of the sample distribution.

Alpha will be set at 0.05 for all analyses, except for the interaction tests which will be evaluated at 0.1 level, and all tests will be two-sided. No adjustment for multiple testing will be performed.

## Discussion

This study will assess the impact of a program of home-based, multi-disciplinary exercise and supportive care on functional exercise capacity, PA levels, muscle strength, HRQoL, anxiety and depression, resilience and symptoms in people with inoperable NSCLC. Our intervention is unique in combining exercise, behavior change support and symptom self-management education. We have chosen exercise capacity as the primary outcome for this study as previous observational work has demonstrated a decline in functional exercise capacity from the point of diagnosis [66] and functional exercise capacity is reported to be associated with outcomes for people with advanced NSCLC, including survival and HRQoL [15, 16]. We are measuring exercise capacity using the submaximal 6MWD, rather than the ‘gold standard’ cardiopulmonary exercise test. This is largely for pragmatic purposes as we anticipate that a number of our study participants will be unable to attend follow-up appointments, especially at the six-month time point, due to a deterioration in their condition. Thus, a proportion of our follow-up measures will be conducted as home-visits. It has previously been reported that there is little change in distance walked between indoor and outdoor walking tracks, so long as the track length is unchanged [69].

Patients with advanced cancer [70], breast cancer [71] and metastatic lung cancer [72] have reported preferences for home-based interventions consisting of aerobic exercise, in the form of walking. A cross-sectional survey of 60 patients with inoperable, metastatic lung cancer reported that exercise is important and that they felt able to complete a light or moderate intensity exercise program and have family to encourage them to exercise. Seventy percent of this group were currently undergoing treatment [72]. Patients receiving palliative care express a desire to exercise, however they cite an inability to attend hospital programs and a lack of mobility or energy as reasons for declining to participate [73].

This study represents a novel, potentially cost-effective, approach to providing multi-disciplinary exercise and supportive care to people with inoperable NSCLC. Home-based interventions have the advantage of reducing participant burden associated with travel, in addition to being relatively low cost. In cancer populations, significant improvements in fatigue are reported following home-based walking programs both during [74, 75] and following chemotherapy or radiotherapy [76]. Previously, a home-based walking and resistance training program in patients with stage IV lung and colorectal cancer demonstrated improvements in patient-reported mobility, fatigue and sleep quality in the intervention group at the completion of an eight-week program [46].

Health behavior change is recognized as a critical component of pulmonary rehabilitation programs [77]. To this end, the intervention implemented in this trial will incorporate several behavioural change health coaching techniques aimed at improving participant self-efficacy and adherence to exercise and PA. These techniques include participant education regarding the health benefits of exercise both in a general sense and in people with cancer, collaborative setting of achievable goals, identification of perceived enablers and barriers to exercise and potential behavioural strategies to overcome barriers, assessing motivational levels for exercise and readiness to change exercise behaviours [36].

The most commonly reported symptoms in individuals with newly diagnosed lung cancer include pain, fatigue, coughing, loss of appetite and sleep disturbance. Those with advanced disease report a high number of uncontrolled symptoms, most notably pain, anorexia and dyspnea, [78], the latter potentially partly relating to underlying chronic lung disease, which is common in patients with lung cancer who have smoked [79]. People may elect to reduce PA levels in an effort to avoid exacerbating symptoms, contributing to the decline in function and muscle strength following lung cancer diagnosis, reported in observational studies [66]. Interventions, such as coaching, to enable symptom self-management targeting symptoms impacting on activity may be an integral component of complex interventions aiming at increasing exercise capacity in patients with inoperable NSCLC.

Longer-term follow up upon completion of exercise intervention is required in both surgical and non-surgical NSCLC populations to assess possible attenuation of benefits gained during exercise programs. Cheville et al. followed a cohort of advanced cancer patients following a three-week program involving eight physiotherapy sessions and found that gains in self-reported physical well-being which were evident in the intervention group at 4 weeks were not maintained at eight and 27-week assessments. Following the eight-week exercise program implemented in this trial, intervention participants will receive a final exercise home-visit to establish a maintenance exercise program. Participants will be supported to continue their exercise program during the maintenance phase with regular scripted exercise phone calls and use of a diary to record monthly exercise adherence until study completion at 6 months. The follow-up intervention in our trial was designed to assist in maintaining any improvements.

## Conclusion

Patients with lung cancer have a poor five-year survival and demonstrate higher burden of disease than those with other forms of cancer. This randomised controlled trial will assess the effects of a multi-disciplinary home-based exercise and supportive care program on physical function, HRQoL and symptoms in people with inoperable NSCLC undergoing treatment. If beneficial, the intervention is designed in a way to enable easy translation into current treatment guidelines for this population.

## Additional file

Additional file 1: Appendix 1. Sample Participant Information and Consent Form. (DOCX 141 kb)

## Abbreviations

6MWD: Six-minute walk distance
ACSM: American college of sports medicine
AQoL: Assessment of quality of life
ATS: American Thoracic Society
BMI: Body mass index
BREQ: Behavioural regulation in exercise questionnaire
CD-RISC: Connor Davidson resilience scale
CFS: Clinical frailty scale
CONSORT: Consolidated standards of reporting trials
ECOG: Eastern Co-operative Oncology Group
ESAS: Edmonton symptom assessment scale
FACT-L: Functional assessment of cancer therapy-lung
FITT: Frequency, intensity, time and type
HADS: Hospital anxiety and depression scale
HRQoL: Health-related quality of life
IPAQ: International physical activity questionnaire
MDASI: MD Anderson symptom inventory
METs: Metabolic equivalents
MID: Minimal important difference
NSCLC: Non-small cell lung cancer
PA: Physical activity
PAAI: Physical activity assessment inventory
RCTs: Randomised controlled trials
REDCap: Research electronic data capture
RM: Repetition maximum
RPE: Rating of perceived exertion
SPIRIT: Standard protocol items: recommendations for interventional trials
SpO2: Peripheral oxygen saturation
TIDier: Template for intervention description and replication
VAS: Visual analogue scale
VO2 peak: Peak oxygen uptake
