# Supplementary material for: Benefits of home-based multidisciplinary exercise and supportive care in inoperable non-small cell lung cancer – protocol for a phase II randomised controlled trial
Source: BMC Cancer. 2017 Sep 29;17:663. doi: 10.1186/s12885-017-3651-4 (PMC5622453; doi:10.1186/s12885-017-3651-4)
Supplement: Additional file 1: Appendix 1. — Sample Participant Information and Consent Form. (DOCX 141 kb) [file 12885_2017_3651_MOESM1_ESM.docx]

*Insert site letterhead*

**Participant Information Sheet/Consent Form**

**Interventional Study** - *Adult providing own consent*

*[Insert site name]*

| **Title** | Benefits of home-based multidisciplinary rehabilitation in non small cell lung cancer |
| --- | --- |
| **Short Title** | *Benefits of home-based exercise in lung cancer* |
| **Protocol Number** | 5.0 |
| **Project Sponsor** | *The National Health and Medical Research Council Australia* |
| **Coordinating Principal Investigator/ Principal Investigator** | Professor Linda Denehy |
| **Associate Investigator(s)** | Professor Sanchia Aranda  Professor Christine McDonald  Associate Professor Meinir Krishnasamy  Associate Professor Louis Irving  Associate Professor Linda Mileshkin  Doctor Ross Clark  Associate Professor Lee Jones  Professor Gary Anderson  Professor Danny Liew  Dr Catherine Granger |
| **Location** | *Insert site name* |

**Part 1 What does my participation involve?**

**1 Introduction**

You are invited to take part in this research project because you have been diagnosed with lung cancer. This research project is testing a new approach to increasing your physical activity levels. This will involve a home-based exercise program suited to your activity goals and include education by a nurse regarding control of any symptoms you may experience.

This Participant Information Sheet/Consent Form tells you about the research project. It explains the tests and treatments involved. Knowing what is involved will help you decide if you want to take part in the research.

Please read this information carefully. Ask questions about anything that you don’t understand or want to know more about. Before deciding whether or not to take part, you might want to talk about it with a relative, friend or your local doctor.

Participation in this research is voluntary. If you don’t wish to take part, you don’t have to. You will receive the best possible care whether or not you take part.

If you decide you want to take part in the research project, you will be asked to sign the consent section. By signing it you are telling us that you:

• Understand what you have read

• Consent to take part in the research project

• Consent to have the tests and treatments that are described

• Consent to the use of your personal and health information as described.

You will be given a copy of this Participant Information and Consent Form to keep.

**2 What is the purpose of this research?**

The purpose of this research project is to compare the effects of home based exercises with usual care in people diagnosed with lung cancer. We also aim to assess factors which act as barriers for you to participate in exercise and activities of daily living.

We currently know that exercise is strongly associated with fitness and well being in many cancer types and has been shown to be safe and practical to perform for people with lung cancer. However, the effects of a home based program to increase physical activity and strength and improve well being in people with lung cancer have not been investigated in Australia. You are being asked to participate in a trial comparing home-based exercise with usual treatment that does not include a specific exercise program.

**3 What does participation in this research involve?**

If you agree to participate in this research you will be participating in a randomised controlled research project. Sometimes we do not know which treatment is best for managing a condition. To find out we need to compare different treatments. We put people into groups and give each group a different treatment. The results are compared to see if one is better. This research project has been designed to make sure the researchers interpret the results in a fair and appropriate way and avoids study doctors or participants jumping to conclusions. To try to make sure the groups are the same, each participant is put into a group by chance (random). There is a one in two chance you will be in the exercise group.

If you agree to participate in this research you will be asked to sign a consent form. We will ask you to sign a separate consent form so we can request information from the Department of Human Services (your Medicare and Pharmaceutical Benefits Scheme claims during the time you are involved in this research). This is so we can understand the financial costs associated with lung cancer. You will be required to come to three assessment appointments at *[insert site name]*. These appointments will take up to one hour and will take place when you start the trial and 9 weeks and 6 months after starting. If you are unable to attend your appointments at 9 weeks and 6 months we will be able to visit you in your home for the appointment if that suits you.

At the assessment appointments we will get you to complete tests that are not part of your normal care. At the first and second appointments you will have a blood test. Trained staff will take about three teaspoons (15mls) of blood from a vein in your arm. The information provided by the blood test will assist us in our assessment of the results of the trial. We will also use an ultrasound to measure the size of your thigh muscles at the first two appointments. Ultrasound measures muscle bulk through the skin by moving the small head of the machine over the muscle for 5 minutes and taking a picture of the muscle (similar to scans in pregnancy). This is a painless technique. Following the first two appointments you will be asked to wear a lightweight activity monitor around your upper arm for a period of seven days, during the day, to measure your physical activity levels.

At each appointment you will be asked to complete two walking tests and fill out a series of questionnaires. This will take approximately one hour. During the walking tests you will be asked to walk up and down a corridor for six minutes while we measure the distance you walk. We will also measure the strength of your thigh muscles and hand grip. At the end of the research project you may be asked to attend a recorded group session to talk about your exercise, activity and symptom levels and how you found the assessments. If you were in the exercise group we will also ask about and how you found the exercises you performed and the nursing symptom management support you received during the project. We can conduct this session in an individual recorded telephone call if you are unable to attend the group session.

We will provide you with information about lung cancer, its treatment and side effects and advice about exercising published by Cancer Council Victoria as part of our usual care.

If you are in the usual care group, our research team will contact you every month for 6 months to see how you are.

In addition to usual care, if you are in the exercise group, you will receive two one-hour physiotherapy sessions in your own home, followed a few days later by a telephone call from our nurse.

Session 1 (week 1, home visit): the physiotherapist and nurse will discuss your current level of exercise and assess your symptoms as well as establish and practice an individual exercise program for you to perform at home for 8 weeks. You will be given an exercise booklet and DVD showing you how to complete your exercises and an exercise diary to complete weekly. We will loan you a mobile phone for 8 weeks and send you a text message once daily reminder to exercise. We will loan you a fitbit so you can keep track of how far you have walked. The nurse will discuss strategies with you to reduce or control your symptoms. Your progress and symptoms will be monitored weekly following this first session by a combination of weekly telephone calls and three home visits during the 8 weeks.

Session 2 (week 9, home visit): with the physiotherapist you will develop an exercise program to continue until you complete the research trial at 6 months. The physiotherapist and nurse will contact you between four and eight times by telephone during this time to talk about your progress.

We will schedule your hospital appointments for the trial to occur with your regular hospital appointments where possible. We can reimburse you for your parking expenses when you attend hospital trial appointments. You will not be paid for your participation in this research.

**4 What do I have to do?**

During this research project there will be no restrictions to your lifestyle or diet. You do not need to alter the medication you are taking. If you are in the exercise group you will be asked to complete a walking and strengthening program in or near your own home.

You will be unable to take part in this study if you have other health problems which prevent you from participating in the exercise.

**5 Other relevant information about the research project**

Ninety-two people will participate in this project which is being conducted by the University of Melbourne. Participants will be recruited from Peter MacCallum Cancer Centre, Royal Melbourne Hospital and Austin Hospital.

This research has been initiated by the study health professionals at each site; Professor Linda Denehy, Professor Sanchia Aranda, Professor Christine McDonald, Associate Professor Louis Irving, Associate Professor Meinir Krishnasamy, Associate Professor Linda Mileshkin and Dr Ross Clark. This research has been funded by the National Health and Medical Research Council of Australia.

**6 Do I have to take part in this research project?**

Participation in any research project is voluntary. If you do not wish to take part, you do not have to. If you decide to take part and later change your mind, you are free to withdraw from the project at any stage.

If you do decide to take part, you will be given this Participant Information and Consent Form to sign and you will be given a copy to keep.

Your decision whether to take part or not to take part, or to take part and then withdraw, will not affect your routine treatment, your relationship with those treating you or your relationship with [insert site name].

**7 What are the alternatives to participation?**

You do not have to take part in this research project to receive treatment at this hospital. Exercise is currently not part of standard care for people with lung cancer. Your study doctor will discuss treatment options with you before you decide whether or not to take part in this research project. You can also discuss the options with your local doctor.

**8 What are the possible benefits of taking part?**

We cannot guarantee or promise that you will receive any benefits from this research; however, possible benefits may include improvements in your fitness, well being, mood, symptoms and treatment side-effects. If the results of the study support the use of home-based exercise this could become part of the standard treatment for people with lung cancer.

**9 What are the possible risks and disadvantages of taking part?**

Participants may feel some muscle discomfort and/or tiredness after exercising. These are generally mild and normal responses to exercise.

The safety and practicality of both exercise tests and exercise in people with lung cancer has been shown in many previous studies. All participants will have a blood test at trial commencement. Participants may be at risk of developing bruising, some discomfort, minor infection or bleeding as a result of this, however these risks are minimal. You should not start exercising if you have a fever and should stop exercising if you have any new chest discomfort or become too breathless to be able to speak. If you are concerned about any new symptoms you can contact any of the staff listed on page 7 below.

If you become upset or distressed as a result of your participation in the research, the study doctor will be able to arrange for counselling or other appropriate support. Any counselling or support will be provided by qualified staff who are not members of the research project team. This counselling will be provided free of charge.

**10 What will happen to my test samples?**

By providing consent you are allowing us to collect a blood sample from you at the start of the trial and at the 9 week assessment. This sample will assist us to determine if there are any changes in markers in the blood that might change if you do more physical activity and that may have an effect on your feelings of well being.

The blood sample collected is for research purposes only and will only be kept for the duration of this project. If you agree, a blood sample will be collected soon after you first agree to participate in this project and at 9 weeks after. All samples collected as part of this research project will be number coded and transferred to the Department of Respiratory Medicine at the Royal Melbourne Hospital for storage. All samples will be stored securely and confidentially in a laboratory freezer. Only members of the research team will have access to the blood samples. All samples will be destroyed at the end of the project under the coordination of Associate Professor Louis Irving according to Melbourne Health protocols. This research project does not involve establishment of a tissue bank.

**11 What if new information arises during this research project?**

Sometimes during the course of a research project, new information becomes available about the treatment that is being studied. If this happens, the research staff will tell you about it and discuss with you whether you want to continue in the research project. If you decide to withdraw, your study doctor will make arrangements for your regular health care to continue.

**12 Can I have other treatments during this research project?**

## Yes, you will have any other treatment that is recommended by your medical team.

13 Are there alternatives to participation?

Your standard care will be continued in the event you choose not to participate. No standard treatment will be withheld at any stage.

**14 What if I withdraw from this research project?**

If you decide to withdraw from this research project, please notify a member of the research team before you withdraw. This notice will allow that person or the research supervisor to inform you if there are any health risks or special requirements linked to withdrawing.

If you do withdraw your consent during the research project, the study doctor and relevant study staff will not collect additional personal information from you, although personal information already collected will be retained to ensure that the results of the research project can be measured properly and to comply with law. You should be aware that data collected by the sponsor up to the time you withdraw will form part of the research project results. If you do not want them to do this, you must tell them before you join the research project.

Any information that we collect will be stored safely in a password protected electronic database and paper records located at [insert site name] and the University of Melbourne in a locked room for seven years. Data will be stored in a way that means you cannot identify which participant it came from. Information will not be released to anyone not involved in the study and may be inspected for purposes of audit by authorised persons only. All data will be destroyed and shredded at the end of the seven years. No further data will be collected after you withdraw from the project.

**15 Could this research project be stopped unexpectedly?**

If this project is terminated before completion we will notify you immediately. This may happen if new information about the risks and benefits of the project become known to the researchers. If this occurs, you will be told about this new information. You will be offered all available care to suit your needs and medical condition.

**16 What happens when the research project ends?**

Once the project is completed, you will be informed of the results via a letter explaining the results. Please ensure we are kept updated with any changes in your address. If you have felt benefit from exercising we will encourage you to continue once the trial is completed. You can discuss this with your local doctor.

**Part 2 How is the research project being conducted?**

**17 What will happen to information about me?**

By signing the consent form you consent to the study doctor and relevant research staff collecting and using personal information about you for the research project. Any information obtained in connection with this research project that can identify you will remain confidential. The information we collect will be stored safely in a password protected electronic data base and paper records located at [insert site name] and the University of Melbourne in a locked filing cabinet and a locked room for seven years. Confidentiality will be maintained by coding the records. Your name will not be kept stored with the records and only researchers involved in the project will have access to the records. At the end of the study all data collected from you will be stored in a way that means you cannot identify who it came from. Information will not be released to anyone not involved in the study and may be inspected for the purposes of audit by authorised persons. At the end of the storage period (seven years), data will be shredded and destroyed.

Any information obtained in connection with this research project that can identify you will remain confidential and will only be used for the purpose of this research project. Your information will only be disclosed with your permission, except as required by law. Information about you may be obtained from your health records held at this, and other, health services for the purposes of this research. By signing the consent form you agree to the study team accessing health records if they are relevant to your participation in this research project. Your health records and any information obtained during the research project are subject to inspection (for the purpose of verifying the procedures and the data) by the relevant authorities and authorised representatives of the Sponsor, the National Health and Medical Research Council, the institution relevant to this Participant Information Sheet, [Name of institution], or as required by law. By signing the Consent Form, you authorise release of, or access to, this confidential information to the relevant study personnel and regulatory authorities as noted above.

It is anticipated that the results of this research project will be published and/or presented in a variety of forums. In any publication and/or presentation, information will be provided as whole group data meaning that you cannot be identified.

**18 How can I access my information?**

In accordance with relevant Australian and Victorian privacy and other relevant laws, you have the right to request access to your information collected and stored by the research team. You also have the right to request that any information with which you disagree be corrected. Please contact the study team member named at the end of this document if you would like to access your information.

**19 Complaints and compensation**

If you suffer any injuries or complications as a result of this research project, you should contact the study team as soon as possible and you will be assisted with arranging appropriate medical treatment. If you are eligible for Medicare, you can receive any medical treatment required to treat the injury or complication, free of charge, as a public patient in any Australian public hospital.

**20 Who is organising and funding the research?**

This research project is being funded by the National Health and Medical Research Council of Australia and conducted by the research investigators lead by Professor Linda Denehy from the University of Melbourne. There will be no financial benefits to you, the study doctors or their institutions from this research project.

**21 Who has reviewed the research project?**

All research in Australia involving humans is reviewed by an independent group of people called a Human Research Ethics Committee (HREC). The ethical aspects of this research project have been approved by the HREC of Peter MacCallum Cancer Centre.

This project will be carried out according to the National Statement on Ethical Conduct in Human Research (2007). This statement has been developed to protect the interests of people who agree to participate in human research studies.

**22 Further information and who to contact**

The person you may need to contact will depend on the nature of your query.

If you want any further information concerning this project or if you have any medical problems which may be related to your involvement in the project (for example, any side effects), you can contact the principal study investigator Professor Linda Denehy on 0418 517 243, or any of the following people:

**Clinical contact person**

| Name | Medical oncology registrar |
| --- | --- |
| Position | [insert site name] |
| Telephone | [insert site switchboard number] and ask to page the Medical Oncology Registrar |

For matters relating to research at the site at which you are participating, the details of the local site complaints person are:

**Complaints contact person**

| Name | Lara Edbrooke |
| --- | --- |
| Position | Trial co-ordinator |
| Telephone | 8344 5377 |
| Email | larae@unimelb.edu.au |

If you have any complaints about any aspect of the project, the way it is being conducted or any questions about being a research participant in general, then you may contact:

| Reviewing HREC name | *Peter MacCallum Cancer Centre* |
| --- | --- |
| HREC Executive Officer |  |
| Telephone | *(03) 9656 1699* |
| Email | [*ethics@petermac.org*](mailto:ethics@petermac.org) |

**Reviewing HREC approving this research** **and HREC Executive Officer details**

**Local HREC Office contact (Single Site - Research Governance Officer)**

| Name | *Insert site Name* |
| --- | --- |
| Position | *Insert site Position* |
| Telephone | *Insert site Telephone* |
| Email | *Insert site email* |

**Consent Form -** *Adult providing own consent*

| **Title** | Benefits of home-based multidisciplinary rehabilitation in non small cell lung cancer |
| --- | --- |
| **Short Title** | *Benefits of home-based exercise in lung cancer* |
| **Protocol Number** | *1.0* |
| **Project Sponsor** | *The National Health and Medical Research Council* |
| **Coordinating Principal Investigator/**  **Principal Investigator** | Professor Linda Denehy |
| **Associate Investigator(s)** | Professor Sanchia Aranda  Professor Christine McDonald  Associate Professor Meinir Krishnasamy  Associate Professor Louis Irving  Associate Professor Linda Mileshkin  Doctor Ross Clark  Associate Professor Lee Jones  Professor Gary Anderson  Ms Janette Gale  Professor Danny Liew  Dr Catherine Granger |
| **Location** | *[insert site name]* |

**Declaration by Participant**

I have read the Participant Information Sheet or someone has read it to me in a language that I understand.

I understand the purposes, procedures and risks of the research described in the project.

I give permission for my doctors, other health professionals, hospitals or laboratories outside this hospital to release information to *[Name of Institution]* concerning my disease and treatment for the purposes of this project. I understand that such information will remain confidential.

I have had an opportunity to ask questions and I am satisfied with the answers I have received.

I freely agree to participate in this research project as described and understand that I am free to withdraw at any time during the study without affecting my future health care.

I understand that I will be given a signed copy of this document to keep.

|  | | | | | | |
| --- | --- | --- | --- | --- | --- | --- |
|  | Name of Participant (please print) | |  |  |  |  |
|  | | | | | | |
|  | Signature |  | | Date |  |  |
|  | | | | | | |

|  | | | | | | |
| --- | --- | --- | --- | --- | --- | --- |
|  | Name of Witness* to Participant’s Signature (please print) | |  | | |  |
|  | | | | | | |
|  | Signature |  | | Date |  |  |
|  | | | | | | |

* Witness is not to be the investigator, a member of the study team or their delegate. In the event that an interpreter is used, the interpreter may not act as a witness to the consent process. Witness must be 18 years or older.

**Declaration by Study Doctor/Senior Researcher^†^**

I have given a verbal explanation of the research project, its procedures and risks and I believe that the participant has understood that explanation.

|  | | | | | | |
| --- | --- | --- | --- | --- | --- | --- |
|  | Name of Study Doctor/  Senior Researcher^†^ (please print) | |  | | |  |
|  | | | | | |  |
|  | Signature |  | | Date |  |  |
|  | | | | | | |

^†^ A senior member of the research team must provide the explanation of, and information concerning, the research project.

Note: All parties signing the consent section must date their own signature.

I consent to the storage and use of blood samples taken from me for use, as described in the relevant section of the Participant Information Sheet, for:

• This specific research project

|  | | | | | | |
| --- | --- | --- | --- | --- | --- | --- |
|  | Name of Participant (please print) | |  | | |  |
|  | | | | | | |
|  | Signature |  | | Date |  |  |
|  | | | | | | |

|  | | | | | | |
| --- | --- | --- | --- | --- | --- | --- |
|  | Name of Witness* to Participant’s Signature (please print) | |  | | |  |
|  | | | | | | |
|  | Signature |  | | Date |  |  |
|  | | | | | | |

* Witness is not to be the investigator, a member of the study team or their delegate. In the event that an interpreter is used, the interpreter may not act as a witness to the consent process. Witness must be 18 years or older.

|  | | | | | | |
| --- | --- | --- | --- | --- | --- | --- |
|  | Name of Study Doctor/  Senior Researcher^†^ (please print) | |  | | |  |
|  | | | | | |  |
|  | Signature |  | | Date |  |  |
|  | | | | | | |

^†^ A senior member of the research team must provide the explanation of and information concerning the research project.

Note: All parties signing the consent section must date their own signature
